# Mesoporous Silica with Different Channel Widths for Cs^+^ Adsorption: A Molecular Dynamics Simulation Study

**DOI:** 10.3390/ma19153278

**Published:** 2026-08-03

**Authors:** Rui Wang, Wensheng Zhang, Jiayuan Ye, Xianquan Wang, Xuguang Zhou

**Affiliations:** 1 State Key Laboratory of Green Building Materials, China Building Materials Academy, Beijing 100024, China; 2 School of Civil Engineering, NingboTech University, Ningbo 315100, China; 3 Zhejiang Provincial Erjian Construction Group Ltd., Ningbo 315100, China; 4 Qian Xuesen Collaborative Research Center of Astrochemistry and Space Life Science, Institute of Drug Discovery Technology, Ningbo 315211, China

**Keywords:** mesoporous silica, molecular dynamics simulation, cesium adsorption, channel size, adsorption mechanism

## Abstract

Mesoporous silica materials have important application prospects in the field of radioactive Cs^+^ adsorption and separation due to their ordered channel structures and high specific surface area. Understanding the microscopic adsorption and diffusion mechanism of Cs^+^ in mesoporous silica channels is key to designing efficient adsorbents. In this study; molecular dynamics simulations were employed to construct mesoporous silica models with channel widths of 2 nm; 4 nm; and 6 nm. The density distribution; radial distribution function (RDF); hydrogen-bonding network; and mean square displacement (MSD) of Cs^+^, NO_3_^−^, and H_2_O in the channels were systematically investigated. The results show that Cs^+^ mainly adsorbs near the channel interface. When the channel width is 2 nm; Cs^+^ is completely captured at the interface; while as the channel size increases; Cs^+^ gradually appears in the center of the channel; indicating weakened adsorption capacity. RDF analysis reveals that approximately 60% of Cs^+^ are within 0.25 nm of the surface –OH groups; forming inner-sphere complexes; while the rest are outer-sphere complexes. The RDF peak of NO_3_^−^ appears at 0.4 nm; confirming its indirect adsorption via electrostatic interaction with Cs^+^. Hydrogen-bonding network analysis shows that the average number of hydrogen bonds per water molecule at equilibrium is about 3.45; which slightly decreases with increasing channel size. MSD results indicate that Cs^+^ has the weakest diffusion capacity; which increases with channel width. This study reveals the regulation mechanism of channel size on Cs^+^ adsorption and diffusion at the atomic level; providing theoretical guidance for the channel engineering design of mesoporous silica-based adsorbents.

## 1. Introduction

The carbon peak and carbon neutrality goals proposed by China provide new opportunities for the vigorous development of nuclear energy. In the steady development of nuclear energy, a large amount of nuclear waste is generated. Among them, ^137^Cs is one of the high-yield and highly hazardous radionuclides from uranium fission in nuclear power plants and spent fuel reprocessing. It is characterized by high heat release, high radioactivity, strong toxicity, high solubility, and high mobility, which pose a serious threat to the environment and public health if improperly disposed [1,2]. Currently, the key to treating radioactive wastewater containing ^137^Cs is volume reduction and solidification. First, radionuclides need to be enriched to meet discharge standards, minimize the volume of concentrated liquid or solid, and improve the decontamination factor. Common methods include chemical precipitation, ion exchange, evaporation concentration, adsorption, and membrane separation [3,4,5,6]. Then, the adsorbent material enriched with radionuclides is solidified to maximize the containment and stabilization of radionuclides. Regarding the enrichment of ^137^Cs in radioactive wastewater, adsorption is favored because of its simple process, low cost, and convenient operation [7,8,9,10]. Therefore, developing high-performance adsorbents and elucidating their microscopic adsorption mechanism have important scientific significance and application value.

### 1.1. Research Status of Mesoporous Silica Adsorbent Materials

Mesoporous silica is a novel inorganic material with a large specific surface area and porous channel structure. It possesses a large number of ordered mesopores with adjustable pore sizes, mainly distributed in the range of 2–50 nm. Research on mesoporous silica can be traced back to the mid-20th century. In 1968, Stöber et al. [11] used ammonia as a catalyst to prepare monodisperse non-porous silica materials. To control particle size and synthesize monodisperse mesoporous silica, Grün [12] and Zhao [13] et al. prepared mesoporous silica microspheres with a narrow particle-size distribution based on Stöber’s method, and they are recognized as pioneers in the development of mesoporous silica materials. In 1990, Yanagisawa et al. [14] reacted NaHSi_2_O_5_·3H_2_O with long-chain alkyltrimethylamine under alkaline conditions to prepare a mesoporous silica material, FSM, with a pore size distribution in the range of 2–4 nm and a specific surface area of about 900 m^2^/g. In 1992, Kresge et al. [15] published the MCM-n series of mesoporous materials in *Nature*, which attracted great attention from the academic community. In 1998, Caruso et al. [16] used a layer-by-layer assembly method to prepare hollow mesoporous silica spheres with a multi-layer composite structure. Subsequently, researchers worldwide developed various methods for preparing hollow mesoporous silica spheres [17,18,19,20]. Currently, mesoporous silica materials are mainly classified into MCM, SBA, HMS, FSM, MSU, and KIT series [21,22,23,24,25,26,27,28].

In the field of radionuclide enrichment and separation, the most widely used mesoporous silica materials are the MCM and SBA series. Štamberg et al. [29] used MCM-41 for uranium adsorption, achieving an adsorption rate of 99% at pH 6. Zhang et al. [30] used SBA-15 for strontium adsorption, and the experimental results showed that the removal rate of strontium by SBA-15 reached 95% within 50 min. Chen et al. [31] compared the uranium adsorption performance of MCM-41 and MCM-48 and found that MCM-48 had better uranium adsorption performance than MCM-41. The main reason was that MCM-48 has finer channels, a larger specific surface area, and better channel connectivity.

Although unmodified mesoporous silica materials have weak surface group activity and are unable to strongly adsorb metal ions in solution, they possess a large number of hydroxyl groups on the surface, which can be utilized for functionalization. The modification methods for mesoporous silica materials are mainly divided into two categories: one is co-condensation synthesis, i.e., one-step synthesis through co-condensation of siloxane and organosiloxane in the presence of a template; the second is post-synthesis, i.e., introducing functional groups through methods such as physical impregnation, chemical grafting, surface deposition, or transition metal substitution after obtaining the mesoporous silica framework [32]. For example, Park et al. [33] loaded NH_4_PHA onto SBA-15 to prepare NH_4_PMA/SBA-15 for cesium nuclide adsorption. The modified SBA-15 exhibited high selective adsorption of cesium in a high-salinity environment. Faghihian et al. [34] used aminopropyl groups to modify MCM-48 for strontium adsorption, and the adsorption capacity of the modified material for strontium reached 5.95 meq/g. Michel et al. [35] loaded potassium copper ferrocyanide nanoparticles onto amino-modified mesoporous silica, achieving rapid cesium adsorption within 5 min. The distribution coefficient K_d,Cs of the modified material in fresh water and seawater reached 10^6^ and 2.0 × 10^5^ mL/g, respectively.

Compared with other mesoporous silica materials, hollow mesoporous silica (HMS) has finer channels, a larger specific surface area, and better channel connectivity. These advantages create more favorable conditions for metal ion transport in the channels and adsorption on the channel surface. The preparation methods of HMS are divided into template methods and etching methods. The template method is widely used because it can effectively control the shape and internal cavity volume of HMS. Template methods are further divided into hard template and soft template methods. Commonly used hard templates include calcium carbonate particles [36] and carbon spheres [37], while soft templates mainly use emulsion droplets [38], vesicles [39], and micelles [40]. When using template methods to prepare HMS, adjusting the morphology and channel structure is very important. The main influencing factors include temperature, stirring mode, inorganic source, precursor, template, reaction medium, pH, and catalyst, among which the choice of inorganic source and template is the most critical. The morphology and channel structure directly affect the mechanical strength, specific surface area, and permeability of HMS. When further modifying HMS, they inevitably affect the loading rate of functional groups, the uniformity of active site distribution, and the diffusion and migration of metal ions in the channels, thereby affecting the adsorption capacity and selectivity of HMS for metal ions.

### 1.2. Molecular Simulation and Its Research Status in the Field of Mesoporous Silica

During nuclear fuel cycle processes, a large amount of low-concentration uranium-containing wastewater is generated, which is radioactive. Experimental research must always be aware of the radioactive impact of uranium wastewater. Moreover, due to experimental limitations, it is difficult to directly observe the adsorption and diffusion of uranium ions in the channels of adsorbents at the atomic level, as well as the chemical reactions at the interface between uranium ions and the adsorbent. To address these issues, new research methods are urgently needed to reveal the adsorption and diffusion mechanisms of uranium ions in channels and at interfaces at the atomic level. Molecular simulation is currently one of the most popular methods for studying the microstructure of materials, offering the advantages of high efficiency, convenience, and safety [41]. The thermodynamic and kinetic information obtained from molecular simulations can be used to analyze and predict the macroscopic properties of the research object, explain reaction mechanisms, and improve the efficiency of material development. Molecular simulation can greatly save time and reduce human and material resource consumption [42].

Molecular simulation obtains the kinetic and thermodynamic information of all particles in the research system through computational calculations, and then analyzes that information to obtain the microscopic and macroscopic properties of the research object [43]. Molecular simulations are divided into quantum mechanical simulations and molecular mechanical simulations based on different theoretical foundations. Molecular mechanical simulations are further divided into Monte Carlo simulations and molecular dynamics simulations [44]. The following sections will introduce the three different molecular simulation methods in detail and compare their similarities, differences, and application scopes.

Quantum chemical calculations mainly focus on the motion of electrons in molecules and the interactions resulting from electron motion. Electron motion is represented by wave functions, and a series of properties such as the structure, conformation, dipole moment, and electron density of molecules in the simulated system can be obtained by directly solving the Schrödinger equation [45]. The simulation results obtained using this quantum chemical calculation are in good agreement with experimental results, but they consume a large amount of computational resources. Under current conditions, only simple systems with no more than a few hundred atoms can be calculated. To improve the computational efficiency of quantum chemical calculations, researchers have advanced the application of quantum mechanics in molecular simulations from two aspects. On one hand, researchers use the adiabatic approximation to separate the motion of nuclei and electrons. For the calculation of multi-electron atoms and molecules, methods such as the Hartree–Fock self-consistent field approximation [46], density functional theory [47], and semi-empirical methods [48] have been proposed. These methods improve the computational efficiency and application range of quantum mechanical simulations while maintaining the required calculation accuracy [49]. On the other hand, researchers have increased the computation speed of computers through various methods, such as developing supercomputers and quantum computers.

Monte Carlo simulation, also known as uncertainty simulation or random simulation, uses the random motion of atoms or molecules in the system and statistical mechanics methods to obtain the statistical and thermodynamic information of the simulated system. The disadvantage of Monte Carlo simulation is that it can only obtain the statistical average of the simulated system and cannot provide the time-dependent dynamic changes in the system [50]. It can provide a series of conformational ensembles formed by the molecules in the simulated system, from which probabilities and related thermodynamic information can be calculated [51]. In summary, Monte Carlo simulation has the advantages of strong adaptability, relatively simple program structure, and relatively time-saving simulation processes. However, because the Monte Carlo method only concerns the distribution and energy of particles in the simulated system and cannot study the motion state of each particle at a given time, it is not suitable for simulating time-dependent ion transport problems in materials.

Molecular dynamics simulation is based on classical mechanics theory and the Born–Oppenheimer approximation principle. It ignores the motion of electrons during the calculation, treats the system’s energy as a function of atomic positions, and writes the interaction parameters between atoms and molecules into force fields. Different force fields are used to describe the interactions between different atoms and molecules. Finally, the particle trajectories and dynamic and thermodynamic statistical information of the system are obtained by solving the classical Newtonian equations of motion, which are used to explain and predict the microscopic and macroscopic properties of the simulated object. Molecular dynamics simulation ignores electron–electron interactions, which greatly reduces computational resource consumption and improves computational efficiency. Although molecular dynamics simulation cannot describe the electronic structure, it can simulate the relevant properties of large systems with high efficiency.

Molecular dynamics methods have been applied to various aspects of mesoporous silica materials. Pajzderska et al. [52] investigated the structure and dynamics of nimodipine confined in a silica matrix under different pore filling degrees using molecular dynamics simulations. The results showed that nimodipine molecules are connected to the silica surface via hydrogen bonds with different lifetimes. The interaction with the silica surface inhibits translational diffusion, while molecules located in the center of the channel exhibit observable, albeit slow, translational diffusion. The silica surface and the presence of additional molecular layers also significantly affect the internal mobility, as reflected by the reorientation of methyl groups and molecular chains. Molecular dynamics simulations provided a unique analytical tool for understanding the effect of filling degree on the structure and dynamics of confined drugs. Lou et al. [53] used molecular dynamics to simulate the cumulative effect of ion displacement cascades in mesoporous silica. The results showed that when the nuclear energy deposition exceeds 30 keV/nm^3^, the volume and structural properties (Si–O–Si bond angle, coordination of O and Si) of mesoporous silica are altered, leading to complete structural collapse. Au^+^ ion irradiation experiments also confirmed similar phenomena: the density of the sample increased with irradiation and saturated after the deposited nuclear energy exceeded 30 keV/nm^3^. Both experiments and simulations indicated that ballistic effects are responsible for the densification of mesoporous silica, and the mechanism can be explained by irradiation-induced sintering. Singh et al. [54] prepared berberine-loaded MCM-41-based mesoporous silica nanoparticles using a modified Stöber method and investigated their role in inhibiting apoptosis. The results showed that the nanoparticles had a particle size of 80–100 nm, uniform distribution, and entrapment efficiency and drug loading of 75.2% and 28.2%, respectively. Drug release was controlled by simple or quasi-diffusion, and molecular dynamics simulations confirmed the drug diffusion behavior. Huang et al. [55] combined the advantages of molecular dynamics in simulating microstructural morphology and lattice dynamics in revealing physical insights to systematically investigate the effect of porous structure on the thermal behavior of mesoporous silica. By analyzing the participation ratio, eigenmode spatial distribution, and mean free path of heat carriers, the heat conduction mechanism was revealed. The results showed that the thermal conductivity of mesoporous silica can be precisely tuned by simultaneously adjusting the porosity, mesopore size, and pore distribution. Yu et al. [56] employed molecular dynamics simulations to study the adsorption and diffusion behavior of Cs^+^ in mesoporous silica channels functionalized with potassium copper ferrocyanide, analyzing the effects of functional group size, solution concentration, and competing cations. The simulation results indicated that Cs^+^ preferentially adsorbs as inner-sphere and outer-sphere surface complexes around the nitrogen atoms of the functional groups. The introduction of competing cations in the solution weakened the interaction between the functional groups and Cs^+^. Wang et al. [57] systematically investigated the adsorption and diffusion behavior of UO_2_^2+^, NO_3_^−^, and H_2_O in hollow mesoporous silica with different pore sizes using molecular dynamics simulations. Guided by the pore-size effects revealed by the simulations, they synthesized hollow mesoporous silica with tunable pore structures and conducted comparative adsorption experiments with MCM-41 and SBA-15. Combined with characterization (XRD, FT-IR, XPS, SEM) and the thermodynamic and kinetic information from simulations, they revealed the adsorption and diffusion mechanisms of U(VI) in mesoporous silica with different pore sizes at the atomic scale.

In summary, although molecular dynamics simulations have been applied to various aspects of mesoporous silica research, systematic investigations focusing on the effect of channel width on Cs^+^ adsorption and diffusion at the atomic scale are still lacking. Existing computational studies have predominantly focused on functionalized materials or specific adsorption systems, while the fundamental role of channel geometry—particularly channel width—on the adsorption behavior of Cs^+^ on pristine silica surfaces remains largely unexplored. The microscopic adsorption configuration, coordination mode, and dynamic evolution of Cs^+^ interactions with surface silanol groups in confined mesoporous environments have yet to be elucidated.

To address these gaps, the present study employs molecular dynamics simulations to systematically investigate the adsorption and diffusion behavior of Cs^+^, NO_3_^−^, and H_2_O in mesoporous silica models with three distinct channel widths (2 nm, 4 nm, and 6 nm). Through comprehensive analysis of density distribution, radial distribution function, hydrogen-bonding network, mean square displacement, and diffusion coefficients, this study aims to elucidate how channel width governs the spatial distribution and interfacial adsorption of Cs^+^, to characterize the coordination modes between Cs^+^ and surface –OH groups, and to quantify the diffusion behavior of ions and water molecules as a function of channel size. The findings are expected to provide theoretical guidance for the rational design of mesoporous silica-based adsorbents with optimized channel structures for efficient Cs^+^ removal. The computational details, including model construction, force field parameters, and simulation protocols, are described in Section 2, followed by the results and discussion in Section 3. Finally, Section 4 presents the conclusions.

## 2. Experimental Methods

This study is a purely molecular dynamics simulation study, rather than an experimental study. The focus of the research lies in revealing the mechanism of how the pore size of mesoporous silica affects the adsorption and diffusion behavior of Cs^+^ at the atomic scale. The mesoporous silica model used is constructed based on a mature and widely accepted method from the literature [53,57]. The detailed process of molecular dynamics simulation is as follows.

### 2.1. Construction of Mesoporous Silica Molecular Models with Different Channel Sizes

To intuitively reveal the adsorption and diffusion mechanisms of Cs^+^, NO_3_^−^, and H_2_O in mesoporous silica channels at the atomic level, and to elucidate the influence of channel size changes on their adsorption and diffusion, Discovery Studio Visualizer was used to establish mesoporous silica–cesium nitrate channel models with different channel widths. The modeling procedure consisted of three steps:

(1) Construction of amorphous SiO_2_ model

**Figure 1:**
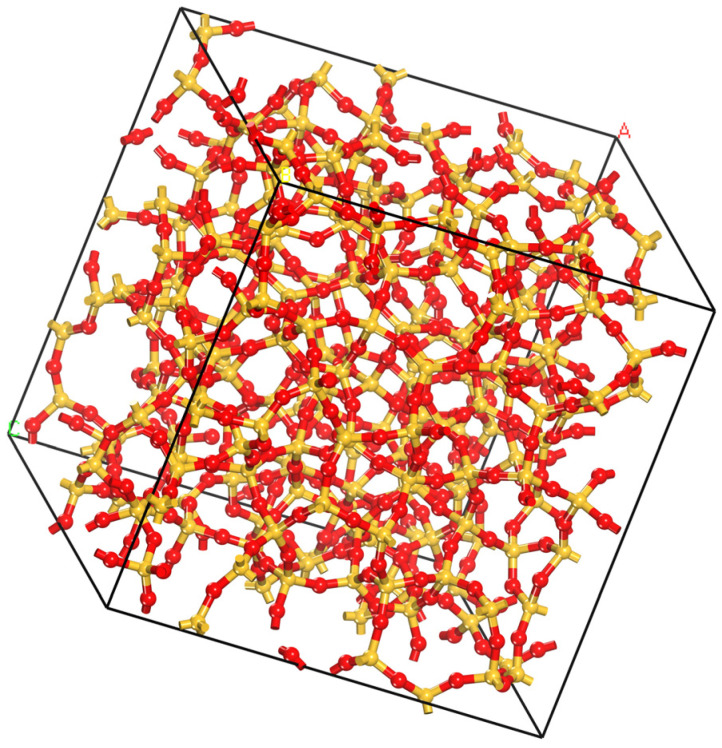
Molecular model of amorphous SiO_2_ (models contain appropriate numbers of atoms for visualization. Red: oxygen; yellow: silicon).

An amorphous SiO_2_ glass molecular structure similar to that of mesoporous silica was selected as the base model. The conjugate gradient method was then used to optimize the structure of the base model to minimize the system energy. Subsequently, the temperature was raised from 300 K to 2500 K at a heating rate of 5 K/ps under the NPT ensemble, and the system was equilibrated at 2500 K for 100 ps. After the heating and equilibration, the system was annealed to 300 K at the same rate and re-equilibrated at 300 K for 100 ps. The simulations used the Nosé–Hoover thermostat and Berendsen barostat, with a time step of 1 fs. The final amorphous SiO_2_ structural model is shown in Figure 1.

(2) Construction of channel models

**Figure 2:**
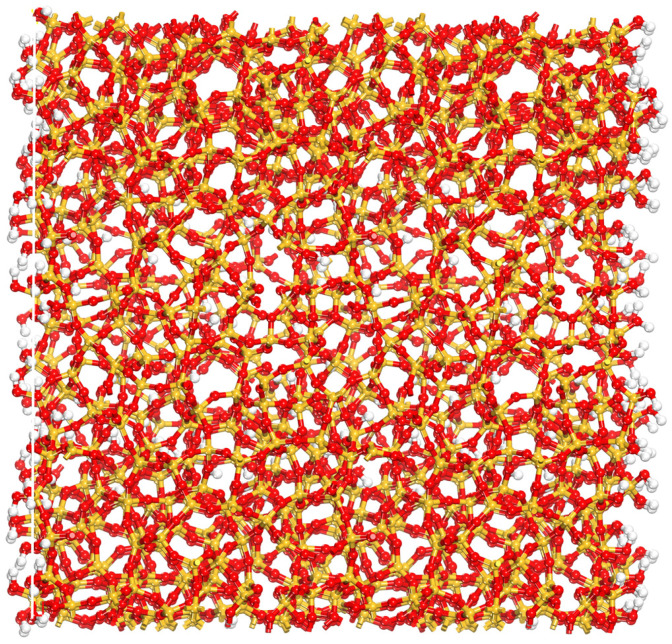
Hydroxylated SiO_2_ molecular model (models contain appropriate numbers of atoms for visualization. Red: oxygen; white: hydrogen; yellow: silicon).

The optimized SiO_2_ structure was cut perpendicular to the [001] crystal plane to obtain mesoporous silica models with channel widths of 2 nm, 4 nm, and 6 nm. Since real mesoporous silica surfaces have a large number of –OH groups, –OH groups were randomly introduced onto the cut channel surface to mimic reality. The resulting structural model is shown in Figure 2.

(3) Solution filling

Based on a simulated wastewater concentration of 0.6 mol/L [10], Cs^+^ and NO_3_^−^ ions were randomly placed in each channel model. Then, the grand canonical Monte Carlo (GCMC) method was used to adsorb water molecules into the channels until a saturated aqueous solution state (density 1 g/cm^3^) was reached. The final model structural parameters are listed in Table 1.

The structural parameters of the mesoporous silica channel models with different channel sizes are summarized in Table 1. The simulation cell dimensions are 4.2 nm × 4.2 nm in the lateral directions (A and B) for all models, while the channel length (C) varies as 9.0 nm, 11.0 nm, and 13.0 nm for the 2 nm, 4 nm, and 6 nm channel widths, respectively. The numbers of Cs^+^ and NO_3_^−^ ions placed in each simulation cell are 57, 70, and 83, corresponding to the three channel sizes. These ion counts were calculated based on a fixed concentration of 0.6 mol/L and the respective cell volumes, ensuring that the Cs^+^ concentration remains identical across all three systems (approximately 0.6 mol/L in each case). The slight differences in ion numbers arise from rounding to the nearest integer.

**Figure 3:**
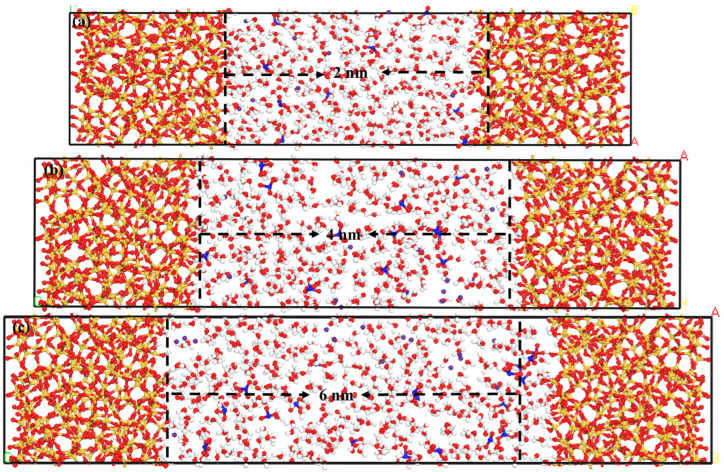
Mesoporous silica molecular model; (**a**) 2 nm channel model; (**b**) 4 nm channel model; (**c**) 6 nm channel model (models contain appropriate numbers of atoms for visualization. Red: oxygen; white: hydrogen; yellow: silicon; dark purple: cesium; dark blue: nitrogen).

The resulting mesoporous silica–cesium nitrate solution molecular models with different channel sizes are shown in Figure 3.

As can be seen from Table 1 and Figure 3, the lateral dimensions (A and B) are fixed at 4.2 nm × 4.2 nm for all models, while the channel length (C) varies from 9.0 nm to 13.0 nm depending on the channel width. In these models, Cs^+^ (dark purple), NO_3_^−^ (dark blue), and H_2_O molecules are distributed within the channels, with the water molecules filling the remaining space to reach a saturated solution state (density of 1 g/cm^3^). The surface –OH groups are introduced on the channel walls to mimic realistic mesoporous silica surfaces.

### 2.2. Molecular Dynamics Simulation Details

All molecular dynamics simulations in this study were performed using the LAMMPS code package. The system contained the mesoporous silica channel model, Cs^+^, NO_3_^−^, and explicit water molecules. The interatomic interaction parameters were all taken from the ClayFF force field, which is particularly suitable for describing the non-bonded interactions of layered silicates, clay minerals, and their interfaces with aqueous solutions, and can reasonably reproduce the long-range electrostatic and van der Waals interactions among silanol groups, soluble ions, and water molecules [58].

The long-range van der Waals interactions in the simulated systems were treated using the Ewald summation method. The long-range electrostatic interactions were treated using the particle-mesh Ewald (PME) method with a grid spacing of about 1.0 Å. The cutoff radius was uniformly set to 12.5 Å, meaning that all pairwise interactions (including electrostatic and van der Waals) are directly calculated when the distance is less than 12.5 Å, and the remainder is compensated via Fourier transform. All simulations were performed under the canonical ensemble (NVT). The temperature was controlled by a Nosé–Hoover thermostat set to 300 K. Since this study focuses on equilibrium properties at constant volume, no pressure control was applied. The time step was set to 1.0 fs to ensure good energy conservation, especially considering the fast vibration of water molecules.

On this basis, a staged relaxation strategy was adopted to systematically transition from a non-equilibrium to an equilibrium state. First, during the initial 500 ps, the channel substrate atoms on both the left and right sides of the mesoporous silica were fixed, allowing only Cs^+^, NO_3_^−^, and H_2_O inside the channels to move freely. This constrained relaxation step aimed to allow ions and water molecules to quickly migrate to the channel surface and form a preliminary adsorption layer without interference from framework thermal noise. Subsequently, all atomic position constraints were removed, and the entire system (including the mesoporous silica framework and the solution) continued to relax for 6000 ps under the NVT ensemble. This stage allowed the thermal vibration of the framework and the motion of ions and water molecules in the solution to fully couple, achieving true equilibrium. The equilibrium criterion was that the standard deviations of the system’s total energy, temperature, and kinetic energy fluctuations over time were less than 0.1% of their respective average values. Finally, the last 3000 ps of the trajectory were used for all subsequent dynamic data analysis to investigate the adsorption and diffusion performance and mechanisms of Cs^+^, NO_3_^−^, and H_2_O in mesoporous silica with different channel sizes.

## 3. Results and Discussion

### 3.1. Density Distribution

**Figure 4:**
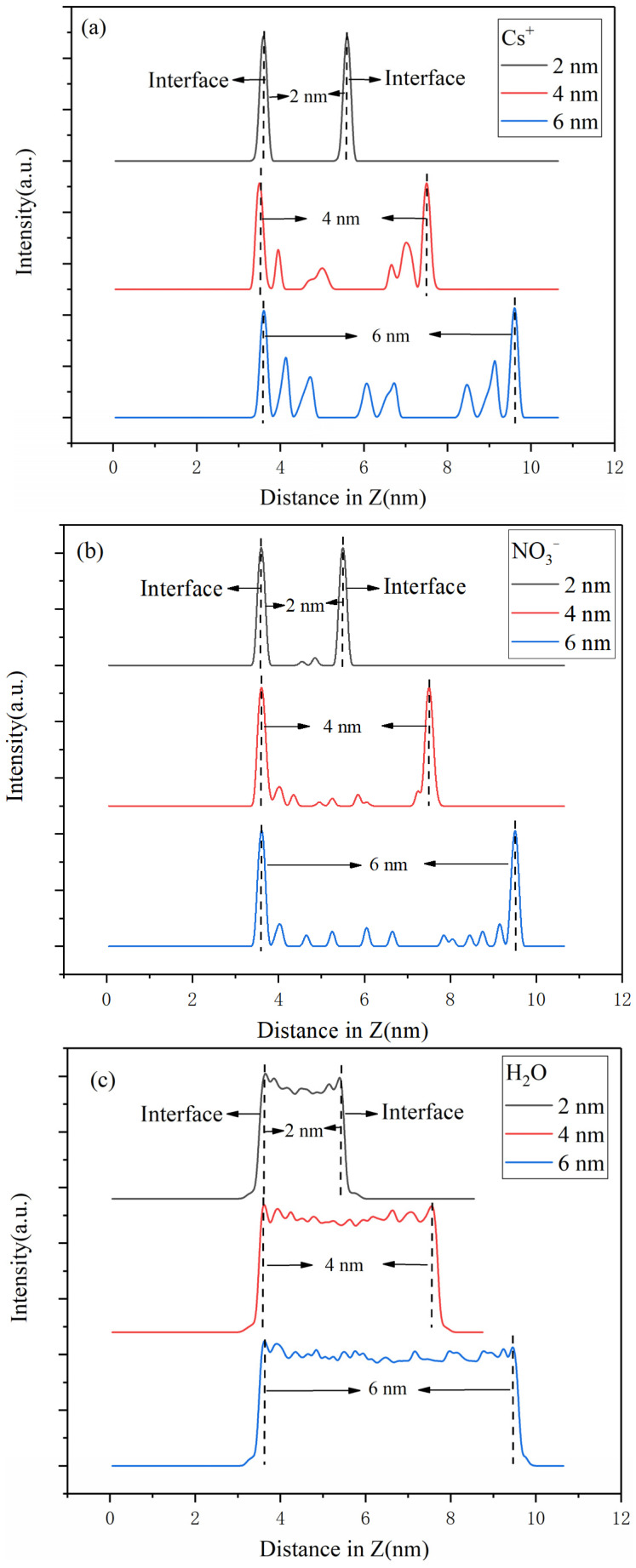
Density profiles of (**a**) Cs^+^, (**b**) NO_3_^−^, and (**c**) H_2_O in mesoporous silica channels with different widths (dashed lines indicate the mesoporous silica interface).

The density distribution profiles of ions and water molecules clearly reflect the distribution of cesium ions, nitrate ions, and water molecules in the mesoporous silica channel models perpendicular to the channel direction. Based on the density distribution results, the adsorption and diffusion patterns of different ions and water molecules at the mesoporous silica channel interface were obtained. The density variation curves of cesium ions, nitrate ions, and water molecules in mesoporous silica models with different channel widths are shown in Figure 4.

Figure 4a shows the density distribution of Cs^+^ in mesoporous silica channels with three different channel widths. It can be clearly observed that sharp density peaks of Cs^+^ appear on both sides, located at the channel interface. This indicates that most Cs^+^ are adsorbed on the mesoporous silica channel surface. Comparing the Cs^+^ density distributions in Figure 3a, it can also be seen that when the channel width is 2 nm, Cs^+^ are all adsorbed on the channel surface, and no Cs^+^ density peaks appear in the middle of the channel. As the channel size increases, the number of Cs^+^ density peaks appearing in the middle of the mesoporous silica channel also shows an increasing trend, indicating that the adsorption capacity of the mesoporous silica channel interface for Cs^+^ weakens with increasing channel size. The main reason for this phenomenon is that the electrostatic attraction of the mesoporous silica channel surface to Cs^+^ distributed in the channel decreases as the channel distance increases. Figure 4b shows the density distribution of NO_3_^−^ in mesoporous silica models with three different channel sizes. It can be seen that NO_3_^−^ is distributed approximately 0.1 nm away from the mesoporous silica channel interface. This indicates that the negatively charged mesoporous silica channel surface first attracts the positively charged Cs^+^, and then Cs^+^ attracts NO_3_^−^ in the aqueous solution through electrostatic attraction, so Cs^+^ and NO_3_^−^ are adsorbed in layers on the mesoporous silica channel surface. Moreover, as the channel width increases, the density distribution of NO_3_^−^ shows a similar variation pattern to that of Cs^+^. This is because the adsorption of NO_3_^−^ strongly depends on Cs^+^. Figure 4c shows the density distribution of H_2_O in mesoporous silica channels with different widths. It can be seen that H_2_O exhibits two density peaks near the mesoporous silica interface that are higher than the bulk water density (1 g/cm^3^), with the peak closest to the mesoporous silica interface being the sharpest. The first density peak of H_2_O is caused by the adsorption of H_2_O via hydrogen bonds to the mesoporous silica. The second density peak is attributed to the layered distribution of hydrated cesium ions and hydrated nitrate ions at the mesoporous silica interface due to the electric double layer effect. These two higher-density water layers can restrict the diffusion of Cs^+^ and NO_3_^−^, making their adsorption more stable on the mesoporous silica surface. Additionally, Figure 3c shows that water density distributions appear within the mesoporous silica framework structures for all three channel widths, indicating that a small number of water molecules adsorb on the mesoporous silica channel surface and then diffuse into the molecular framework.

### 3.2. Radial Distribution Function (RDF)

**Figure 5:**
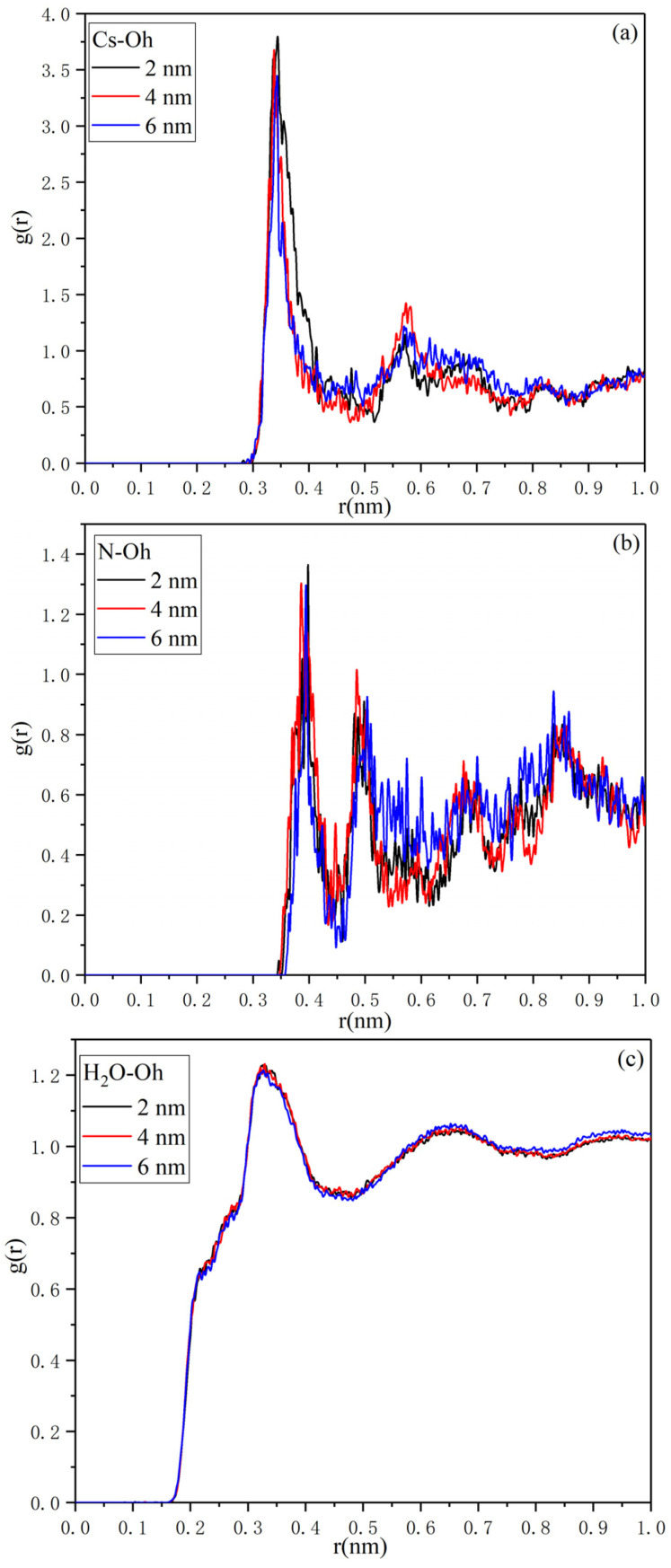
RDF of (**a**) Cs^+^, (**b**) NO_3_^−^, and (**c**) H_2_O with surface hydroxyl groups of mesoporous silica channels.

The position of the first sharp peak in the RDF curve indicates the distance between two atomic pairs. If the distance is less than the bond length, it can be considered that a bond forms between the two atoms. Figure 5 shows the RDF curves of cesium ions, nitrate ions, and water molecules with the hydroxyl groups on the mesoporous silica channel surface for different channel widths.

Figure 5a–c, it can be seen that the change in mesoporous silica channel size has little effect on the adsorption of Cs^+^, NO_3_^−^, and H_2_O at the channel interface. Cs^+^ and H_2_O have a sharp RDF peak at about 0.35 nm, but the intensity of the Cs^+^ RDF peak is much higher than that of H_2_O. The results is consistent with previous MD simulations of Cs^+^ adsorption on silica surfaces and clay minerals, where Cs^+^–O distances typically fall in the range of 0.28–0.33 nm for inner-sphere complexes [10,58]. This indicates that the interaction between Cs^+^ and the surface –OH groups is stronger, and Cs^+^ are distributed within a shorter range of the –OH groups. By calculating the RDF between each Cs^+^ and the surface hydroxyl groups of the mesoporous silica channels for the three different channel widths, it was found that the first peak of the Cs–OH RDF appears near 0.25 nm for about 60% of the pairs. This indicates that Cs^+^ adsorbed on the mesoporous silica channel surface may undergo two types of adsorption. When the Cs–O distance is ≤0.25 nm, Cs^+^ form monodentate or multidentate coordination with the surface –OH, indicating that Cs^+^ are adsorbed on the mesoporous silica channel surface via inner-sphere adsorption. When the Cs–OH distance is longer than the Cs–O bonding distance, Cs^+^ do not coordinate with the surface hydroxyl groups but are adsorbed via electrostatic interaction (outer-sphere adsorption). Figure 5b shows the RDF curves between NO_3_^−^ and the surface –OH groups. The first sharp peak appears at about 0.4 nm, which is larger than the distance between Cs^+^ and the surface –OH. This result indicates that the hydroxyl groups first adsorb Cs^+^, and then Cs^+^ attract NO_3_^−^ via electrostatic interactions. Furthermore, from Figure 5, the intensity of the NO_3_^−^–OH RDF peak is lower than that of Cs^+^–OH but higher than that of H_2_O–OH, indicating that the interaction between surface hydroxyl groups and water molecules is very weak.

### 3.3. Hydrogen-Bonding Network

Based on the geometric criterion for hydrogen bonds, the average number of hydrogen bonds per water molecule in mesoporous silica channels with different widths was calculated at 300 ps, 4000 ps, and 5000 ps. The statistical results are shown in Table 2.

Table 2 shows that at 300 ps, the average number of hydrogen bonds per water molecule in the 2 nm, 4 nm, and 6 nm channels is 2.86, 2.82, and 2.79, respectively. At this stage, the system is at the initial stage of molecular dynamics simulation, with Cs^+^, NO_3_^−^, and H_2_O randomly distributed in the mesoporous silica channels. The ions and water molecules have not fully hydrated, and the adsorption of Cs^+^, NO_3_^−^, and H_2_O by the mesoporous silica channel interface has not yet occurred. Therefore, the average number of hydrogen bonds per water molecule is basically the same. When the simulation time increases to 4000 ps, the average number of hydrogen bonds per water molecule in the three different channel widths increases to 3.46, 3.45, and 3.43, respectively. This is because after a period of molecular dynamics simulation, Cs^+^, NO_3_^−^, and H_2_O have reached an adsorption equilibrium state in the mesoporous silica channels. From the previous analysis, Cs^+^, NO_3_^−^, and H_2_O are adsorbed in layers on the mesoporous silica surface, and a high-density solution layer appears on the surface. These factors lead to a higher probability for the hydrogens in water molecules to form hydrogen bonds with oxygen atoms from nitrate ions, siloxane tetrahedral units, and surface hydroxyl groups. Therefore, the average number of hydrogen bonds per water molecule increases with simulation time. Additionally, from Table 2, at 4000 ps, the average number of hydrogen bonds per water molecule shows a decreasing trend with increasing mesoporous silica channel size. The reason for this phenomenon is that the increased channel width weakens the adsorption capacity of the mesoporous silica channel surface for Cs^+^, NO_3_^−^, and H_2_O. Table 2 also shows the average number of hydrogen bonds per water molecule at 5000 ps, which is almost unchanged from the values at 4000 ps. This is because after the system reaches adsorption equilibrium, the adsorption state of Cs^+^, NO_3_^−^, and H_2_O on the mesoporous silica channel surface maintains a dynamic equilibrium, so the average number of hydrogen bonds per water molecule remains essentially constant.

### 3.4. Mean Square Displacement (MSD) and Diffusion Coefficients

**Figure 6:**
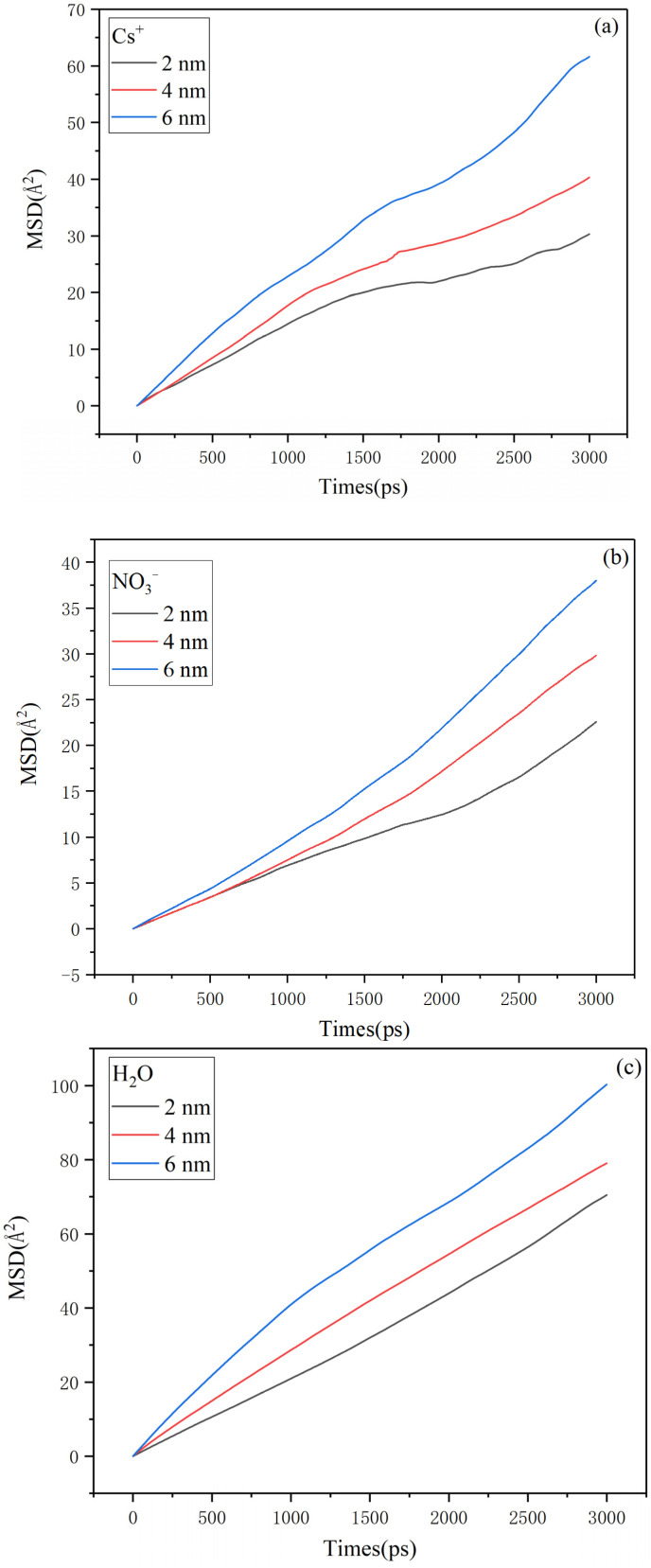
MSD of (**a**) Cs^+^, (**b**) NO_3_^−^, and (**c**) H_2_O in mesoporous silica channels of different sizes.

In molecular dynamics simulations, the mean square displacement (MSD) is used to describe the change in particle displacement over time. The diffusion ability of Cs^+^, NO_3_^−^, and H_2_O in mesoporous silica channels of 2 nm, 4 nm, and 6 nm can be expressed by MSD, and the results are shown in Figure 6.

From Figure 6a–c, it can be seen that the diffusion abilities of Cs^+^, NO_3_^−^, and H_2_O in the mesoporous silica channels are different. At any time during the molecular dynamics simulation, Cs^+^ has the weakest diffusion ability, followed by NO_3_^−^, while H_2_O has the strongest diffusion ability. This is because Cs^+^ and NO_3_^−^ undergo hydration in the aqueous solution to form hydrated ions. Since the binding ability of different ions with water molecules varies greatly, the diffusion abilities of Cs^+^, NO_3_^−^, and H_2_O differ significantly. Moreover, comparing the diffusion abilities of ions and water molecules in the three different channel widths in Figure 6, it can be observed that as the channel width increases, the diffusion abilities of Cs^+^, NO_3_^−^, and H_2_O also increase. There are two reasons for this phenomenon. First, the increased channel width provides larger space for Cs^+^, NO_3_^−^, and H_2_O to diffuse. Second, as the channel size increases, the adsorption capacity of the channel interface for some of the Cs^+^, NO_3_^−^, and H_2_O in the channel weakens.

Additionally, the diffusion coefficients of Cs^+^, NO_3_^−^, and H_2_O were calculated using the Einstein relation $D=\frac{1}{6}{lim}_{t\to \infty }\frac{d}{dt}\mathrm{MSD}(t)$. The results are summarized in Table 3.

The diffusion coefficients listed in Table 3 directly describe the diffusion capabilities of Cs^+^, NO_3_^−^, and H_2_O in mesoporous silica with different channel widths. The diffusion coefficients of Cs^+^ obtained from molecular dynamics simulations are consistent with a previous study [58]. Table 3 shows that in the same channel, Cs^+^ exhibits the weakest diffusion ability, followed by NO_3_^−^, while H_2_O has the strongest diffusion ability. This is because Cs^+^ forms strong inner-sphere complexes with the surface –OH groups on the channel walls, and its larger hydration radius, combined with stronger electrostatic confinement at the channel interface, results in the most significant restriction on migration. NO_3_^−^ is indirectly adsorbed at the channel interface via Cs^+^ bridging, with relatively weak direct interaction with the surface, showing moderate diffusion ability. In contrast, H_2_O molecules, due to their small size and weak hydrogen-bonding interactions with the surface, possess the highest mobility [52,56]. Furthermore, Table 3 reveals that as the mesoporous silica channel width increases from 2 nm to 6 nm, the diffusion coefficients of Cs^+^, NO_3_^−^, and H_2_O all gradually increase, indicating that in wider channel structures, ions and water molecules experience weaker interfacial confinement, favoring more free migration. This trend is consistent with the density distribution and MSD analyses, further confirming the role of channel confinement effects in regulating the transport behavior of ions and molecules.

## 4. Conclusions

In this study, molecular dynamics simulations were used to systematically investigate the adsorption and diffusion behavior of Cs^+^, NO_3_^−^, and H_2_O in mesoporous silica models with channel widths of 2 nm, 4 nm, and 6 nm. The main conclusions are as follows:

(1) Channel width significantly affects the adsorption distribution of Cs^+^.

In the narrow 2 nm channel, Cs^+^ are completely adsorbed at the mesoporous silica channel interface. As the channel size increases, some Cs^+^ migrate to the center of the channel, and the interface adsorption capacity weakens, indicating that narrow channels are more favorable for Cs^+^ capture. This effect is attributed to the enhanced physical confinement and interfacial electrostatic attraction in narrower channels.

(2) Cs^+^ adsorbs in a mixed coordination mode.

About 60% of Cs^+^ form inner-sphere complexes with the –OH groups on the mesoporous silica channel surface, while the rest form outer-sphere electrostatic complexes. NO_3_^−^ is indirectly adsorbed via Cs^+^ bridging at about 0.4 nm from the interface. Changes in channel size do not alter the ion adsorption mode, indicating that the coordination chemistry is primarily governed by the surface –OH groups.

(3) Interfacial water molecules form a stable hydrogen-bonding network.

The average number of hydrogen bonds per water molecule at equilibrium is about 3.45, which is slightly higher in the narrow channel, contributing to the stable adsorption of Cs^+^. The stability of the hydrogen-bonding network decreases slightly with increasing channel size, reflecting the loosening effect of reduced confinement on the interfacial water structure.

(4) Cs^+^ has the weakest diffusion capacity, which is regulated by channel size.

The MSD of Cs^+^ is much lower than that of H_2_O and NO_3_^−^. As the channel width increases, the diffusion capacity of all three species increases, confirming that wider mesoporous silica channels lead to less stable ion adsorption and easier migration. The enhanced diffusion capacity is directly attributed to the enlarged channel space and weakened interfacial constraints.

In summary, the simulation results of this study demonstrate that channel size regulates the spatial distribution and diffusion behavior of ions through the physical confinement effect, while the surface –OH groups determine the coordination mode and binding strength of ions. These two factors work synergistically to dictate the overall adsorption efficiency of Cs^+^ in mesoporous silica channels. Narrow-channel mesoporous silica achieves efficient and stable adsorption of Cs^+^ by enhancing interfacial electrostatic attraction and the hydrogen-bonding network. This study reveals the regulation mechanism of channel size on Cs^+^ adsorption and diffusion behavior at the atomic level, providing a clear channel engineering strategy for the design of high-performance mesoporous silica-based cesium adsorbents.

## Figures and Tables

**Table 1:** Structural parameters of mesoporous silica channel models with different channel sizes.

Channel Size (nm)	Cs^+^	NO_3_^−^	Simulation Cell Parameters (nm)
2	57	57	A = 4.2, B = 4.2, C = 9.0
4	70	70	A = 4.2, B = 4.2, C = 11.0
6	83	83	A = 4.2, B = 4.2, C = 13.0

**Table 2:** Average number of hydrogen bonds per water molecule in mesoporous silica channels with different widths.

Times (ps)	2 nm	4 nm	6 nm
300	2.86	2.82	2.79
4000	3.46	3.45	3.43
5000	3.46	3.45	3.43

**Table 3:** Diffusion coefficients of Cs^+^, NO_3_^−^ and H_2_O in different pore sizes of mesoporous silica.

Species	2 nm Channel	4 nm Channel	6 nm Channel
Cs^+^	0.48 × 10^−10^	0.86 × 10^−10^	1.24 × 10^−10^
NO_3_^−^	1.52 × 10^−10^	2.43 × 10^−10^	3.36 × 10^−10^
H_2_O	4.87 × 10^−10^	6.35 × 10^−10^	7.92 × 10^−10^

## Data Availability

The original contributions presented in this study are included in the article. Further inquiries can be directed to the corresponding author.
